# Robust response to pembrolizumab plus lenvatinib in a patient with renal cell carcinoma with rhabdoid features: A case report

**DOI:** 10.1016/j.eucr.2025.103140

**Published:** 2025-07-23

**Authors:** Shinkuro Yamamoto, Satoshi Fukata, Takashi Karashima, Atsushi Kurabayashi, Rie Yoshimura, Kaoru Furihata, Yuhei Shiba, Tomoya Nao, Hirofumi Satake, Keiji Inoue

**Affiliations:** a Department of Urology, Kochi Medical School, Nankoku, 783-8505, Japan; b Department of Urology, Kochi Prefectural Aki General Hospital, Aki, 784-0027, Japan; c Department of Pathology, Kochi Medical School, Nankoku, 783-8505, Japan; d Department of Pathology, Chikamori Hospital, Kochi, 780-8522, Japan

**Keywords:** Renal cell carcinoma, Rhabdoid feature, Pembrolizumab, Lenvatinib

## Abstract

Renal cell carcinoma (RCC) with rhabdoid features is a rare and highly aggressive condition with no established treatment for early recurrence. We herein report a case involving a 58-year-old man who underwent nephrectomy for clear-cell RCC with rhabdoid features and developed rapid metastases within 3 months. Treatment with pembrolizumab plus lenvatinib achieved a sustained partial response, except for vertebral metastasis. Thereafter, radiotherapy was added, and systemic therapy resumed, which maintained disease control for 2 years. Immunohistochemistry revealed partial PD-L1 expression in rhabdoid areas. This case highlights the potential efficacy of pembrolizumab plus lenvatinib in RCC with rhabdoid features.

## Highlights

• RCC with rhabdoid features is a rare and highly aggressive subtype.
• Studies on treatments for early recurrence of RCC with rhabdoid features are scant.
• Pembrolizumab plus lenvatinib achieved robust control of RCC with rhabdoid features.

## 1 Introduction

Studies show that rhabdoid features in renal cell carcinoma (RCC) can serve as histological markers of dedifferentiation associated with highly aggressive clinical behavior and poor prognosis.^1^^,^^2^ Such cases, which can be classified as WHO/ISUP grade 4, are relatively rare, with limited data regarding optimal therapeutic approaches. Although immune checkpoint inhibitors (ICIs) have demonstrated efficacy in advanced RCC,^3^ their role in RCC with rhabdoid features have not been well established. Here, we report a case involving early postoperative recurrence of clear cell RCC with rhabdoid differentiation that was successfully controlled with pembrolizumab plus lenvatinib combination therapy.

## 2 Case presentation

**Fig. 1:**
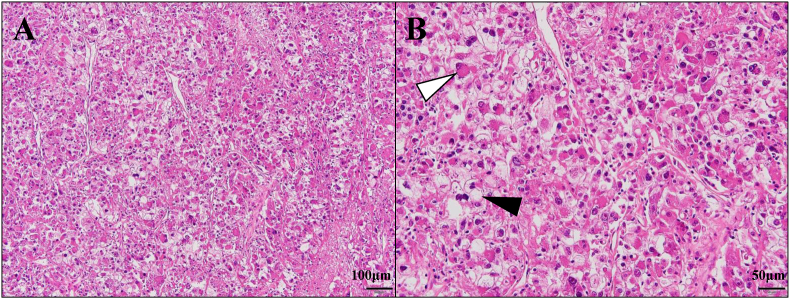
Hematoxylin and eosin (H&E) staining of renal cell carcinoma with rhabdoid features. A: Low-power view ( × 100) showing tumor cells with solid growth pattern. B: High-power view ( × 200) revealing two distinct components: typical clear cell carcinoma morphology with abundant clear cytoplasm (black arrow) and rhabdoid-like features characterized by eccentrically located nuclei and eosinophilic intracytoplasmic inclusion body-like structures (white arrow).

**Fig. 2:**
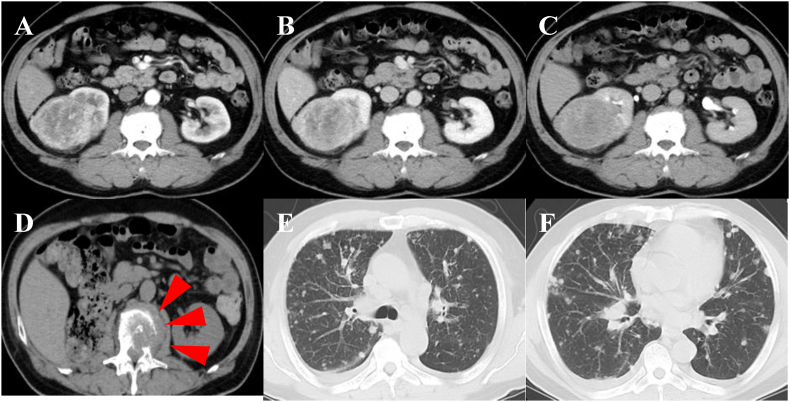
Computed tomography (CT) images of the abdomen and chest. A–C: Contrast-enhanced CT prior to surgery showing a right renal mass. A: 30 s. B: 70 s. C: 300 s. D: CT image of L2 bone metastasis upon the diagnosis of postoperative recurrence (red arrows). E, F: CT images of multiple lung metastases upon the diagnosis of postoperative recurrence.

A 58-year-old man underwent laparoscopic radical nephrectomy for a left renal mass initially staged as cT2cN0M0. Histopathological examination revealed clear cell RCC with rhabdoid differentiation at a pathological stage of pT3a (Fig. 1, Fig. 2A–C). Postoperative imaging at 2 months showed no evidence of recurrence. However, by the third month, the patient presented with fever and displayed rapid progression of metastatic disease involving the lungs, adrenal glands, and multiple bones (Fig. 2D, E, F), for which he was classified as “poor risk” according to the IMDC criteria and referred to our institution.

**Fig. 3:**
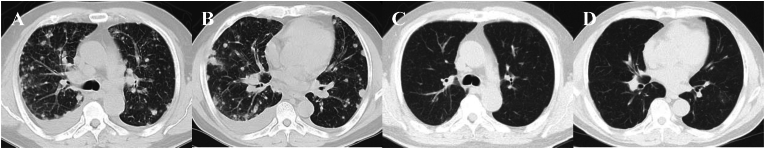
Computed tomography (CT) images of the chest. A, B: CT images of multiple lung metastases obtained 3 days after the diagnosis of postoperative recurrence revealing rapid progression. C, D: CT images taken 3 months after initiating combination therapy with pembrolizumab and lenvatinib showing the almost complete disappearance of the rapid progression in lung lesions.

Considering the aggressive disease course (Fig. 3A and B), combination therapy with pembrolizumab (200 mg) and lenvatinib (20 mg) was initiated on day 1. Although computed tomography (CT) on day 14 showed progressive disease, pembrolizumab was continued until day 22. Remarkably, a follow-up CT on day 28 demonstrated a partial response, with substantial regression of lung metastases and a decrease in adrenal and most bone lesions. However, progression of the L2 vertebral lesion raised concern for potential neurological complications.

As such, systemic therapy was paused, and external beam radiotherapy (25 Gy in 5 fractions) was administered to the L2 lesion between days 38 and 42. Combination therapy was then resumed on day 48. CT performed 3 months after treatment initiation confirmed partial response (Fig. 3C and D). At the 2-year follow-up, imaging tests confirmed sustained near-critical response, with no new metastatic lesions having been detected.

During treatment, several adverse events were observed. Grade 3 hypertension developed on day 7, which was managed with 2.5 mg of amlodipine. On day 55, the patient experienced Grade 3 vomiting, which prompted a reduction in the lenvatinib dose from 20 to 14 mg. Moreover, Grade 3 hand–foot syndrome occurred on day 77, necessitating temporary interruption of lenvatinib. After the patient's symptoms had resolved by day 80, lenvatinib was reintroduced at 10 mg on day 87. The adjusted regimen was well tolerated thereafter, with no further significant adverse events or disease progression.

## 3 Discussion

The PD-1 inhibitor pembrolizumab and multi-kinase inhibitor lenvatinib have shown promising efficacy in advanced RCC. Lenvatinib, a multitargeted kinase inhibitor of FGFR1–4, VEGFR1-3, KIT, RET, and PDGFR-β,^4^ has been proven effective for liver and thyroid cancers. The CLEAR study on advanced RCC emphasized the efficacy of combination therapy consisting of pembrolizumab and lenvatinib. Motzer et al. reported that the median progression-free survival (95 % CI) was 23.9 months (95 % CI, 20.8 to 27.7) with lenvatinib plus pembrolizumab and 9.2 months (95 % CI, 6.0 to 11.0) with sunitinib (HR, 0.47 [95 % CI, 0.38 to 0.57]). They also reported that the objective response rate also favored the combination over sunitinib (71.3 % v 36.7 %; relative risk 1.94 [95 % CI, 1.67 to 2.26]).^5^ Another study reported that the ORR for metastatic bone tumors was 64.7 % for lenvatinib plus pembrolizumab and 22.7 % for sunitinib.^6^ In cases wherein the tumor generally shrinks while receiving lenvatinib plus pembrolizumab and an increase in metastatic bone tumor developed in a few areas, as in our case, external radiation therapy may be provided to improve long-term prognosis after appropriate drug withdrawal. Only few studies have reported on the use of ICIs as part of combination therapy for RCC with rhabdoid features.

Rhabdoid changes in RCC have been associated with an aggressive clinical course and a poor prognosis, with an overall survival ranging from 8 to 46 months, which can be attributed to presentation at an advanced stage. In fact, the largest combined series that included 232 patients found that 76 %–91 % of patients presented with pT3 or pT4 disease. Metastasis has been found to occur in up to 70 % of cases, with cancer-specific mortality rates ranging from 40 % to 64 %. The sites for distant metastasis reported to date include the lungs, bone, liver, adrenal glands, and diaphragm.^1^

**Fig. 4:**
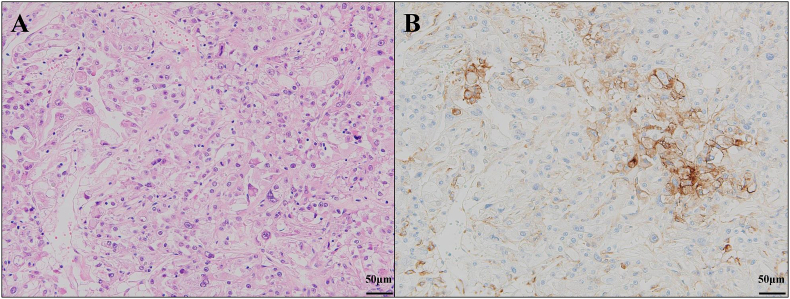
A: Hematoxylin and eosin (H&E) staining showing solid growth of tumor cells. B: Immunohistochemical staining for PD-L1. PD-L1 expression was observed in the area exhibiting rhabdoid features.

Previous studies have highlighted the importance of evaluating PDL-1 expression in RCCs with sarcomatoid tumors via immunohistochemistry (IHC). After directly comparing two PD-L1 antibodies, Joseph et al. found that 89 % of the tested RCCs with sarcomatoid differentiation showed concordance in terms of PD-L1 positivity and that 50 % (13/26) of RCCs with sarcomatoid differentiation displayed coexpression of PD-L1 on neoplastic cells and contained PD-1-positive tumor-infiltrating lymphocytes.^7^ Using a semiquantitative method, namely the H-score (percentage of positive cells showing a membranous staining pattern [0–100] × staining intensity [0–3]), Kawakami et al. found that 118 patients with sarcomatoid RCC and 92 patients with non-sarcomatoid clear cell RCC were positive for PD-L1 expression.^8^ Thus, PDL-1 expression may be used as a predictor of response to combination therapy with ICIs in sarcomatoid RCC. In RCC with rhabdoid features (e.g., sarcomatoid RCC), PDL-1 expression is upregulated and may be a predictor of therapeutic response. In the present case, IHC staining for PDL-1 revealed PDL-1 positivity in areas where the rhabdoid changes were moderate (Fig. 4A and B). In contrast, IHC staining for PDL-1 came back negative in some areas where the rhabdoid changes were strong. Although determining the correlation between rhabdoid changes and PDL-1 expression based on solely on this case may prove difficult, the fact that PDL-1 was expressed at least to some extent supports the hypothesis that PD-1 inhibitors can be effective as evidenced by the significant response to the pembrolizumab plus lenvatinib combination therapy.

## 4 Conclusion

This case report demonstrates the potential efficacy of pembrolizumab plus lenvatinib in the treatment of RCC with rhabdoid features. PD-L1 expression may serve as a biomarker for predicting response to ICIs in such aggressive variants. Integrating local radiotherapy into systemic therapy may further enhance long-term control.

## Informed consent and patient details

Appropriate informed consent was obtained from the patient.

## CRediT authorship contribution statement

**Shinkuro Yamamoto:** Conceptualization, Data curation, Visualization, Writing – original draft, Writing – review & editing. **Satoshi Fukata:** Conceptualization, Data curation, Supervision, Writing – review & editing. **Takashi Karashima:** Conceptualization, Data curation, Supervision, Writing – review & editing. **Atsushi Kurabayashi:** Data curation, Visualization, Writing – review & editing. **Rie Yoshimura:** Writing – review & editing. **Kaoru Furihata:** Data curation, Visualization, Writing – review & editing. **Yuhei Shiba:** Writing – review & editing. **Tomoya Nao:** Writing – review & editing. **Hirofumi Satake:** Writing – review & editing. **Keiji Inoue:** Supervision, Writing – review & editing.

## Data statement

All data supporting the findings of this study are available within the paper.

## Declaration of generative AI and AI-assisted technologies in the writing process

The authors declare that no generative AI or AI-assisted technologies were used in the writing or editing of this manuscript.

## Funding sources

This research did not receive any specific grant from funding agencies in the public, commercial, or not-for-profit sectors.

## Declaration of competing interests

The authors declare no conflict of interest.

## References

[1] Adeniran A.J.S.B., Humphrey P.A. (2024; Jul 1). Sarcomatoid and rhabdoid renal cell carcinoma: clinical, pathologic, and molecular genetic features. Am J Surg Pathol.

[2] Hahn A.W., Lebenthal J., Genovese G., Sircar K., Tannir N.M., Msaouel P. (2022). The significance of sarcomatoid and rhabdoid dedifferentiation in renal cell carcinoma. Cancer Treat Res Commun.

[3] Grünwald V.P.T., Eto M., Kopyltsov E. (2023). Corrigendum: phase 3 CLEAR study in patients with advanced renal cell carcinoma: outcomes in subgroups for the lenvatinib-plus-pembrolizumab and sunitinib arms. Front Oncol.

[4] Tohyama O., Matsui J., Kodama K. (2014). Antitumor activity of lenvatinib (e7080): an angiogenesis inhibitor that targets multiple receptor tyrosine kinases in preclinical human thyroid cancer models. J Thyroid Res.

[5] Motzer R.J., Porta C., Eto M. (2024). Lenvatinib plus pembrolizumab versus sunitinib in first-line treatment of advanced renal cell carcinoma: final prespecified overall survival analysis of CLEAR, a phase III study. J Clin Oncol.

[6] Grunwald V., Powles T., Eto M. (2023). Corrigendum: phase 3 CLEAR study in patients with advanced renal cell carcinoma: outcomes in subgroups for the lenvatinib-plus-pembrolizumab and sunitinib arms. Front Oncol.

[7] Joseph R.W., Millis S.Z., Carballido E.M. (2015). PD-1 and PD-L1 expression in renal cell carcinoma with sarcomatoid differentiation. Cancer Immunol Res.

[8] Kawakami F., Sircar K., Rodriguez-Canales J. (2017). Programmed cell death ligand 1 and tumor-infiltrating lymphocyte status in patients with renal cell carcinoma and sarcomatoid dedifferentiation. Cancer.

